# Phenotypic Spectrum at Diagnosis of Age‐Related Endotypes of Type 1 Diabetes Mellitus: A Cross‐Sectional Study in China

**DOI:** 10.1111/1753-0407.70111

**Published:** 2025-06-12

**Authors:** Qiaoli Zhou, Xueqin Zheng, Chenguang Ma, Wei Gu

**Affiliations:** ^1^ Department of Endocrinology Children's Hospital of Nanjing Medical University Nanjing China

**Keywords:** β‐cell autoimmunity, endotype, phenotypic variation, population heterogeneity, Type 1 diabetes mellitus

## Abstract

**Background:**

Emerging evidence suggests the presence of distinct endotypes of Type 1 diabetes mellitus (T1DM): T1DE1 in individuals diagnosed at age < 7 years in contrast to T1DE2 in those diagnosed at ≥ 13 years of age. We aimed to comprehensively explore the phenotypic heterogeneity of T1DM with respect to the age‐related endotypes.

**Methods:**

This cross‐sectional study was conducted in China and involved 1204 children newly diagnosed with T1DM who were admitted to the pediatric department of a tertiary hospital from January 1, 2010 to December 31, 2023. The patients were divided into three age groups: < 7 years (T1DE1), 7–12 years, and ≥ 13 years (T1DE2). A comparison was made among the age groups regarding demographic characteristics, glucose metabolism, β‐cell autoimmunity, and metabolic decompensation.

**Results:**

Patients under 7 years exhibited a shorter symptom duration before diagnosis, along with the lowest fasting and postprandial C‐peptide and C‐peptide to glucose ratio levels and the highest postprandial glucose levels. They also showed the highest insulin autoantibody positivity rate and creatine kinase‐MB levels. In contrast, patients aged 13 and older had the highest HbA1c levels and glutamate decarboxylase antibody positivity rate. In addition, this group showed the highest prevalence of TPOAb and TgAb positivity, as well as the largest proportion of abnormal liver function cases.

**Conclusions:**

The study illustrates age‐specific phenotypic heterogeneity in pediatric T1DM, indicating the presence of distinct endotypes. Further investigation of these endotypes may offer more evidence for the precise treatment of T1DM.

## Graphical abstract

Emerging evidence points to distinct endotypes of T1DM: T1DE1 (diagnosed < 7 years) and T1DE2 (diagnosed ≥ 13 years). To explore phenotypic heterogeneity in Type 1 diabetes mellitus (T1DM) with respect to age‐related endotypes. A cross‐sectional study of 1204 newly diagnosed T1DM children in China (2010–2023), divided into three age groups: T1DE1, 7–12 years, and T1DE2. Age‐specific phenotypic heterogeneity in pediatric T1DM indicates distinct endotypes, suggesting potential for precise treatment strategies.

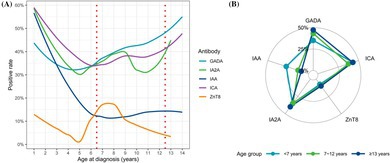

**Summary**

- A cross‐sectional study in China investigated 1204 children with T1DM between 2010 and 2023.
- Phenotypic heterogeneity was observed at the onset of T1DM, showing age‐specific variations.
- The study explored various factors, including demographics, glucose metabolism, β‐cell function, β‐cell autoimmunity, and metabolic decompensation.
- The findings indicated specific disease endotypes associated with different age groups, emphasizing the critical role of age‐related factors in both diagnosis and management of T1DM.

## 1 Introduction

Type 1 diabetes mellitus (T1DM) is a chronic autoimmune disease characterized by the destruction of the insulin‐producing pancreatic β cells, leading to a loss of insulin secretion and subsequent hyperglycemia. The incidence of T1DM is estimated to be 15 cases per 100 000 individuals, with a prevalence of 9.5% [1]. Furthermore, there has been an increase in the incidence of T1DM over time. T1DM is a highly heterogeneous disease, evident through substantial variations in epidemiology, etiopathogenesis, clinical progression, and responses to interventions [2]. The pathogenesis of the disease may result from a combination of genetic predisposition and environmental factors. However, the disease process is still not well understood, leading to suboptimal prevention and treatment. The current classification schema inadequately captures the heterogeneity or guides specific treatment strategies [3]. A potential method to advance precision in management is through the identification of disease endotypes, which are distinguished by specific underlying mechanisms or pathophysiological processes [2, 4]. Although the concept of T1DM endotypes is still debated, accumulating data supports their existence. Age is the most significant factor contributing to the heterogeneity of T1DM, with profound impacts on the epidemiology, risk, and progression [5]. The incidence of T1DM peaks between the ages of 5 and 9, with a second peak occurring near puberty [6]. Overall, young children tend to be more greatly influenced, potentially due to a mix of immunologic, metabolic, and genetic factors [7]. Two distinct endotypes of T1DM, T1DE1 and T1DE2, have recently been described based on pancreas histopathology and characterized by age at diagnosis (at cut‐offs of 7 and 13 years) [8, 9]. T1DE1 is characterized by its early onset, specifically in children diagnosed with T1DM under 7 years old. It is distinguished by a few residual insulin‐containing islets with aberrant proinsulin processing and a high presence of immune infiltrates dominated by CD8^+^ T cells and CD20^+^ B cells [9]. Clinically, it correlates with a rapid decline in C‐peptide after diagnosis [10], the “early IAA first” seroconversion, and the presence of HLA‐DR4/DQ8 haplotype [11]. In contrast, T1DE2 is characterized by a diagnosis later in life (≥ 13 years old), a higher percentage of residual insulin‐containing islets with preserved proinsulin processing, and a reduced presence of immune infiltrates [9]. Clinically, it is associated with a slower decline in C‐peptide levels after diagnosis, the “late GADA first” seroconversion, and the expression of HLA‐DR3/DQ2 [7]. The two endotypes, representing a more aggressive versus a less aggressive T1DM, align with the two peaks of incidence observed at different ages of diagnosis in childhood and adolescence [4]. Regarding intervention, T1DE1 may respond better to immunotherapy that targets specific immune cell subsets, such as rituximab or teplizumab, while T1DE2 may respond better to GAD‐alum therapy [2].

Previous research on T1DE1 and T1DE2 was mostly based on the histopathological analysis of pancreatic samples [8, 9]. Few epidemiological studies have explored the phenotypic manifestations of T1DM endotypes. In these studies, individuals with T1DM were divided into age groups based on age at diagnosis: < 7 years (T1DE1), 7–12 years, and ≥ 13 years (T1DE2). One study analyzed data from the UK Genetic Resource Investigating Diabetes cohort. The results showed that patients with T1DE1 had a lower C‐peptide level at the time of diagnosis, progressed more rapidly toward total C‐peptide loss, and had minimal β‐cell retention [12]. Another study analyzed data from the Finnish Pediatric Diabetes Register to examine and compare various demographic, clinical, autoimmune, and genetic characteristics across different age groups. The findings revealed substantial age‐related differences in most of the characteristics analyzed [13].

However, evidence of gaps still exists. Evidence for glucose metabolism, thyroid function, and other organ functions across age groups was generally lacking. Moreover, racial/ethnic disparities have been observed in patients with T1DM [14, 15], and evidence is lacking for other racial/ethnic groups. The aim of the current study was to comprehensively explore the phenotypic heterogeneity of T1DM with respect to age‐related endotypes in an Asian population. Characteristics of demographics, glucose metabolism, β‐cell autoimmunity, metabolic decompensation, thyroid function, and liver function of a population of Chinese children and adolescents with newly diagnosed T1DM were compared among age groups to characterize their distinct phenotypes.

## 2 Materials and Methods

### 2.1 Study Population

A cross‐sectional retrospective study was conducted on 1639 consecutive children newly diagnosed with diabetes mellitus at the Endocrinology Department of Children's Hospital of Nanjing Medical University between January 1, 2010 and December 31, 2023. Cases of Type 2 diabetes mellitus (T2DM) and other specific types of diabetes were excluded. Among the 1235 patients newly diagnosed with T1DM, 1204 were enrolled in the study cohort, while the remaining 31 cases were excluded due to missing or conflicting key information, as shown in Figure S1. The study was reviewed and approved by Children's Hospital of Nanjing Medical University (approval number: 202405001‐1) and conducted in accordance with the principles outlined in the Declaration of Helsinki.

### 2.2 Definition of Characteristics

All relevant data were collected at the time of diagnosis. The patients were divided into age groups based on age at diagnosis: < 7 years (T1DE1), 7–12 years, and ≥ 13 years (T1DE2). The calendar year for birth and diagnosis was divided into four seasons according to common practice in Southern China: Spring (March–May), summer (June–August), autumn (September–November), and winter (December–February). The duration of symptoms before diagnosis was identified from the chief complaints and was categorized into groups of ≤ 1 week, 1–4 weeks, and > 4 weeks. Based on the practice guideline in China [16], diabetic ketosis (DK) was defined as having hyperglycemia (serum glucose ≥ 11.1 mmol/L) and positive ketones (serum β‐hydroxybutyrate ≥ 3 mmol/L or ketonuria). Diabetic ketoacidosis (DKA) was defined if a DK patient had a pH < 7.3 or serum HCO_3_ < 15 mmol/L. The cut‐off values for laboratory tests were determined based on the local standard or practice.

### 2.3 Autoantibodies

Diabetes‐related autoantibodies analyzed in this study included glutamate decarboxylase antibody (GADA), insulin autoantibody (IAA), islet cell antibody (ICA), islet antigen 2 antibody (IA2A), and zinc transporter 8 antibody (ZnT8). Between 2010 and June 2019, GADA and IAA were detected using radioimmunoassay, with a transition to the chemiluminescence method in July 2019 for the detection of GADA, IAA, and ICA. Subsequently, since October 2022, the chemiluminescence method has been utilized to detect GADA, IAA, ICA, IA2A, and ZnT8.

### 2.4 Assessment of Pancreatic β‐Cell Function

Evaluation of islet function involved the steamed bread meal test (SBMT). During the SBMT, participants underwent an overnight fast, with the initial blood sample collected after fasting considered the baseline (0 min) measurement. Subsequently, individuals consumed steamed bread prepared with N flour (age < 5 years, *N* = 50 g; ages 5–10 years, *N* = 75 g; age > 10 years, *N* = 100 g) within a 10‐min window. Blood samples were drawn at 0, 60, and 120‐min post‐ingestion to assess blood glucose, insulin, and C‐peptide levels.

### 2.5 Statistical Analysis

For laboratory tests, values beyond the detection limits were replaced by the detection limits. The missing values were not imputed. Categorical variables were presented as frequency/total (%). Continuous variables were presented as the mean ± standard deviation (SD) or median (interquartile range [IQR]), as appropriate. To test for independence of age groups and patient characteristics, we used Pearson's chi‐square test for categorical variables and the Kruskal–Wallis test for continuous variables. Post hoc pairwise comparisons were conducted using Pearson's chi‐squared test for categorical variables and the Wilcoxon rank‐sum test for continuous variables, with corrections for multiple testing using the false discovery rate. All statistical analyses were performed using the R software (version 4.1.2). All statistical tests were two‐sided. Statistical significance was set at *p* < 0.05. Plots were generated using the R package “ggplot2” [17].

## 3 Results

### 3.1 Study Population

**FIGURE 1:**
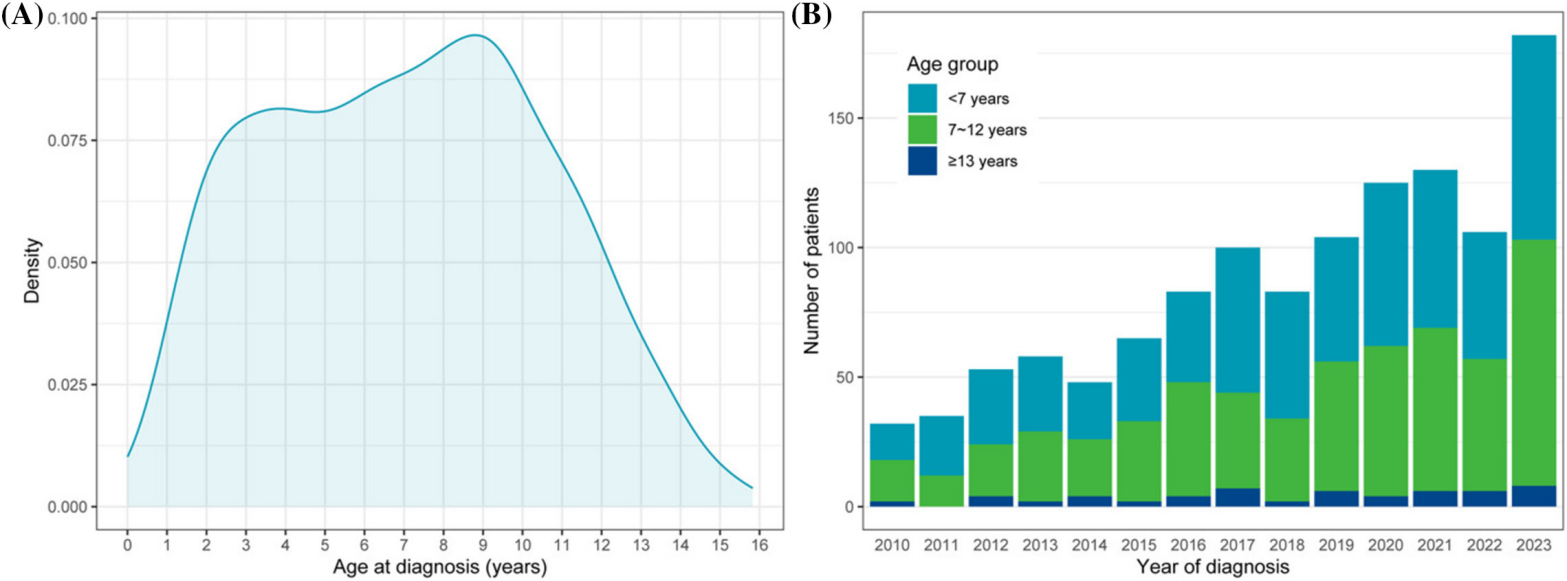
Distribution of age at diagnosis (A) and age groups across different years of diagnosis (B).

Our study population included 1204 patients with T1DM aged 0–16 years at diagnosis. The distribution of age at diagnosis is shown in Figure 1a. Two peaks were observed: one at 3–4 years old and the other at 9 years old. The number of patients diagnosed each year is shown in Figure 1b, showing an increasing trend. Most patients belong to the age groups of < 7 years (589; 49%) and 7–12 years (558; 46%), which is consistent across different years of diagnosis (shown in Figure 1b).

The baseline characteristics of the study population are shown in Table 1 and were compared by age group in Table 2. Among all patients, 577 (48%) were male. The most common season of diagnosis was spring (29%), whereas the season of birth was evenly distributed. The duration of symptoms before diagnosis was mostly within 4 weeks (81%). Significant differences were observed, where patients < 7 years of age at diagnosis had the shortest duration of symptoms.

**TABLE 1:** Characteristics of the study population.

Characteristic	Missing	Overall (*N* = 1204)
Sex	0 (0%)	
Male		577/1204 (48%)
Female		627/1204 (52%)
Age at diagnosis (years)	0 (0%)	7.09 ± 3.51
< 7		589/1204 (49%)
7–12		558/1204 (46%)
≥ 13		57/1204 (5%)
BMI SDS at diagnosis	335 (28%)	−0.84 (−1.79, 0.13)
Duration of symptoms (weeks)	411 (34%)	
≤ 1		170/793 (21%)
1–4		474/793 (60%)
> 4		149/793 (19%)
Season of diagnosis	0 (0%)	
Spring		355/1204 (29%)
Summer		286/1204 (24%)
Autumn		284/1204 (24%)
Winter		279/1204 (23%)
Season of birth	0 (0%)	
Spring		308/1204 (26%)
Summer		304/1204 (25%)
Autumn		304/1204 (25%)
Winter		288/1204 (24%)
Birth weight (kg)	50 (4%)	3.41 ± 0.61
GADA positive	50 (4%)	455/1154 (39%)
ICA positive	152 (13%)	426/1052 (40%)
IA2A positive	993 (82%)	74/211 (35%)
ZnT8 positive	993 (82%)	18/211 (8%)
IAA positive	55 (5%)	229/1149 (20%)
TPOAb positive	197 (16%)	134/1007 (13%)
TgAb positive	65 (5%)	101/1139 (9%)
DK	3 (0%)	989/1201 (82%)
DKA	3 (0%)	625/1201 (52%)
HbA1c (%)	48 (4%)	12.27 ± 2.44
< 7.0		22/1156 (2%)
7.0–14		922/1156 (80%)
> 14		212/1156 (18%)
Serum glucose (mmol/L)	389 (32%)	
Fasting		7.54 ± 2.53
2‐h		22.07 ± 4.71
Serum C‐peptide (nmol/L)	25 (2%)	
Fasting		0.11 (0.07, 0.17)
2‐h		0.32 (0.22, 0.45)

Abbreviations: BMI, body mass index; DK, diabetic ketosis; DKA, diabetic ketoacidosis; DM, diabetes mellitus; GADA, glutamate decarboxylase antibody; HbA1c, hemoglobin A1c; IA2A, islet antigen 2 antibody; IAA, insulin autoantibody; ICA, islet cell antibody; SDS, standard deviation score; TgAb, thyroglobulin antibody; TPOAb, thyroid peroxidase antibody; ZnT8, zinc transporter 8 antibody.

**TABLE 2:** Characteristics of the study population by age group.

Characteristic	< 7 years (*N* = 589)	7–12 years (*N* = 558)	≥ 13 years (*N* = 57)	*p*			
				Among age groups	< 7 vs. ≥ 13 years	< 7 vs. 7–12 years	7–12 vs. ≥ 13 years
General characteristics							
Male	304/589 (52%)	248/558 (44%)	25/57 (44%)	0.043	0.399	0.052	> 0.9
BMI SDS at diagnosis	−0.82 (−1.81, 0.12)	−0.85 (−1.78, 0.16)	−0.71 (−1.50, −0.05)	0.9	0.751	0.751	0.751
Duration of symptoms (weeks)				0.004	0.176	0.003	0.509
≤ 1	97/385 (25%)	64/370 (17%)	9/38 (24%)				
1–4	234/385 (61%)	221/370 (60%)	19/38 (50%)				
> 4	54/385 (14%)	85/370 (23%)	10/38 (26%)				
Glucose metabolism							
HbA1c (%)	11.69 ± 2.08	12.77 ± 2.66	13.34 ± 2.11	< 0.001	< 0.001	< 0.001	0.03
				< 0.001	0.011	0.001	0.893
< 7.0	14/562 (2%)	7/543 (1%)	1/51 (2%)				
7.0–14	488/562 (87%)	398/543 (73%)	36/51 (71%)				
> 14	60/562 (11%)	138/543 (25%)	14/51 (27%)				
Serum glucose (mmol/L)							
Fasting	7.58 ± 2.53	7.47 ± 2.49	7.81 ± 2.91	0.8	> 0.9	> 0.9	> 0.9
2‐h	22.94 ± 5.06	21.32 ± 4.15	20.55 ± 4.68	< 0.001	0.003	< 0.001	0.161
Serum C‐peptide (nmol/L)							
Fasting	0.10 (0.06, 0.14)	0.14 (0.08, 0.21)	0.16 (0.11, 0.22)	< 0.001	< 0.001	< 0.001	0.023
2‐h	0.26 (0.18, 0.37)	0.38 (0.28, 0.53)	0.40 (0.31, 0.53)	< 0.001	< 0.001	< 0.001	0.16
CGR (×10^−6^)							
Fasting	12.64 (8.87, 19.20)	17.06 (11.73, 26.13)	22.27 (15.45, 30.09)	< 0.001	0	0	0.024
2‐h	10.51 (6.80, 16.86)	16.31 (11.79, 24.06)	19.25 (14.93, 27.16)	< 0.001	0	0	0.065
β‐Cell autoimmunity							
GADA positive	196/559 (35%)	233/540 (43%)	26/55 (47%)	0.011	0.105	0.022	0.556
ICA positive	218/505 (43%)	187/498 (38%)	21/49 (43%)	0.2	> 0.9	0.22	0.836
IA2A positive	33/93 (35%)	37/108 (34%)	4/10 (40%)	> 0.9	> 0.9	> 0.9	> 0.9
ZnT8 positive	7/93 (8%)	10/108 (9%)	1/10 (10%)	0.9	> 0.9	> 0.9	> 0.9
IAA positive	154/559 (28%)	70/535 (13%)	5/55 (9%)	< 0.001	0.005	0.001	0.527
Metabolic decompensation							
DK	488/588 (83%)	451/556 (81%)	50/57 (88%)	0.4	0.456	0.456	0.456
DKA	315/588 (54%)	281/556 (51%)	29/57 (51%)	0.6	> 0.9	0.9	> 0.9
pH	7.22 ± 0.24	7.25 ± 0.17	7.20 ± 0.19	0.2	0.45	0.258	0.258
HCO_3_ (mmol/L)	13.28 ± 6.82	14.15 ± 6.40	13.29 ± 7.29	0.15	0.864	0.156	0.766
β‐Hydroxybutyrate (mmol/L)	3.27 ± 2.47	3.23 ± 2.50	3.59 ± 2.21	0.7	0.685	0.685	0.685
Ketonuria	224/352 (64%)	212/326 (65%)	24/34 (71%)	0.7	0.748	0.748	0.748
Thyroid function							
TPOAb positive	48/486 (10%)	75/472 (16%)	11/49 (22%)	0.004	0.016	0.016	0.313
TgAb positive	26/555 (5%)	67/529 (13%)	8/55 (15%)	< 0.001	0.012	0.001	0.834
TSH (μIU/mL)				0.001	0.019	0.027	0.019
< 0.2	12/558 (2%)	13/533 (2%)	5/55 (9%)				
> 5	49/558 (9%)	74/533 (14%)	3/55 (5%)				
0.2–5	497/558 (89%)	446/533 (84%)	47/55 (85%)				
Liver function							
ALT (U/L)				< 0.001	0.001	0.021	0.044
< 5	8/366 (2%)	13/366 (4%)	2/37 (5%)				
> 40	4/366 (1%)	16/366 (4%)	5/37 (14%)				
5–40	354/366 (97%)	337/366 (92%)	30/37 (81%)				
AST (U/L)				0.14	0.241	0.581	0.437
< 5	0/366 (0%)	2/366 (1%)	1/37 (3%)				
> 40	23/366 (6%)	23/366 (6%)	3/37 (8%)				
5–40	343/366 (94%)	341/366 (93%)	33/37 (89%)				
ALP (U/L)				< 0.001	0.001	0.048	0.012
< 143	21/370 (6%)	38/368 (10%)	9/38 (24%)				
> 406	22/370 (6%)	25/368 (7%)	6/38 (16%)				
143–406	327/370 (88%)	305/368 (83%)	23/38 (61%)				
Others							
CK‐MB > 25 U/L	175/369 (47%)	130/368 (35%)	13/38 (34%)	0.003	0.195	0.003	> 0.9

Abbreviations: ALP, alkaline phosphatase; ALT, alanine aminotransferase; AST, aspartate aminotransferase; BMI, body mass index; CGR, c‐peptide to glucose ratio; CK‐MB, creatine kinase‐MB; DK, diabetic ketosis; DKA, diabetic ketoacidosis; DM, diabetes mellitus; GADA, glutamate decarboxylase antibody; HbA1c, hemoglobin A1c; IA2A, islet antigen 2 antibody; IAA, insulin autoantibody; ICA, islet cell antibody; SDS, standard deviation score; TgAb, thyroglobulin antibody; TPOAb, thyroid peroxidase antibody; TSH, thyroid‐stimulating hormone; ZnT8, zinc transporter 8 antibody.

### 3.2 Glucose Metabolism

**FIGURE 2:**
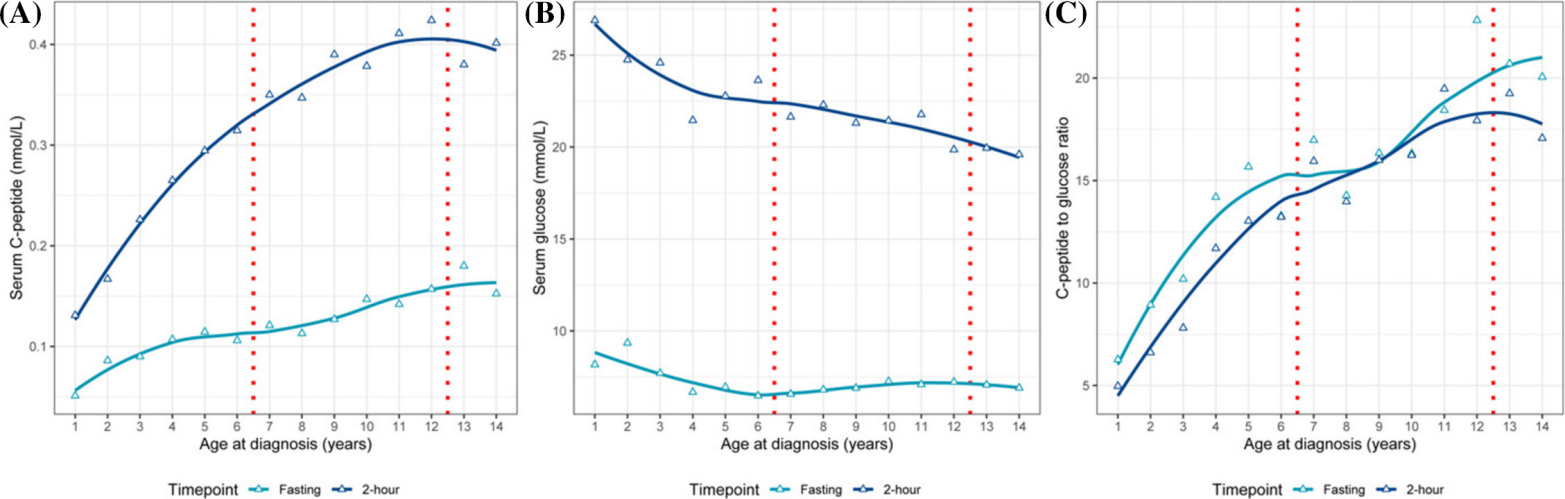
Glucose metabolism parameters across different ages at diagnosis: serum C‐peptide (A); glucose (B); and C‐peptide to glucose ratio (C). The color indicates the timepoint of the assessments. Median values were plotted as triangles. The smoothed lines were fitted by local polynomial regression. Red dotted lines denote the boundaries between age groups.

We analyzed the glucose metabolism parameters across different ages at diagnosis, including serum C‐peptide, glucose, and C‐peptide to glucose ratio (CGR). As shown in Figure 2, all parameters exhibited an age‐related trend. Both fasting and 2‐h C‐peptide showed an increasing trend with age, with a greater increase for 2‐h C‐peptide. Two‐hour glucose showed a monotonic decreasing trend with a steeper slope before the age of five. Fasting glucose decreased before the age of six and then fluctuated within a small range. For CGR, both fasting and 2‐h, increasing trends with age were observed. As shown in Table 2, the comparison of the parameters between age groups showed that C‐peptide, both fasting and 2‐h, was significantly lower in patients < 7 years of age compared to patients in other age groups. CGR showed a similar pattern. Patients < 7 years of age also had the highest 2‐h glucose. Fasting glucose was similar among groups. Notably, post hoc pairwise comparison showed no significant differences between the 7–12 years and ≥ 13 years age groups except for fasting CGR (*p* = 0.024). For HbA1c, patients < 7 years of age had the lowest HbA1c level (11.69% ± 2.08%), which is significantly lower than the other age groups (12.77% ± 2.66% and 13.34% ± 2.11% for patients 7–12 and ≥ 13 years of age, respectively; both *p* < 0.001).

### 3.3 β‐Cell Autoimmunity

**FIGURE 3:**
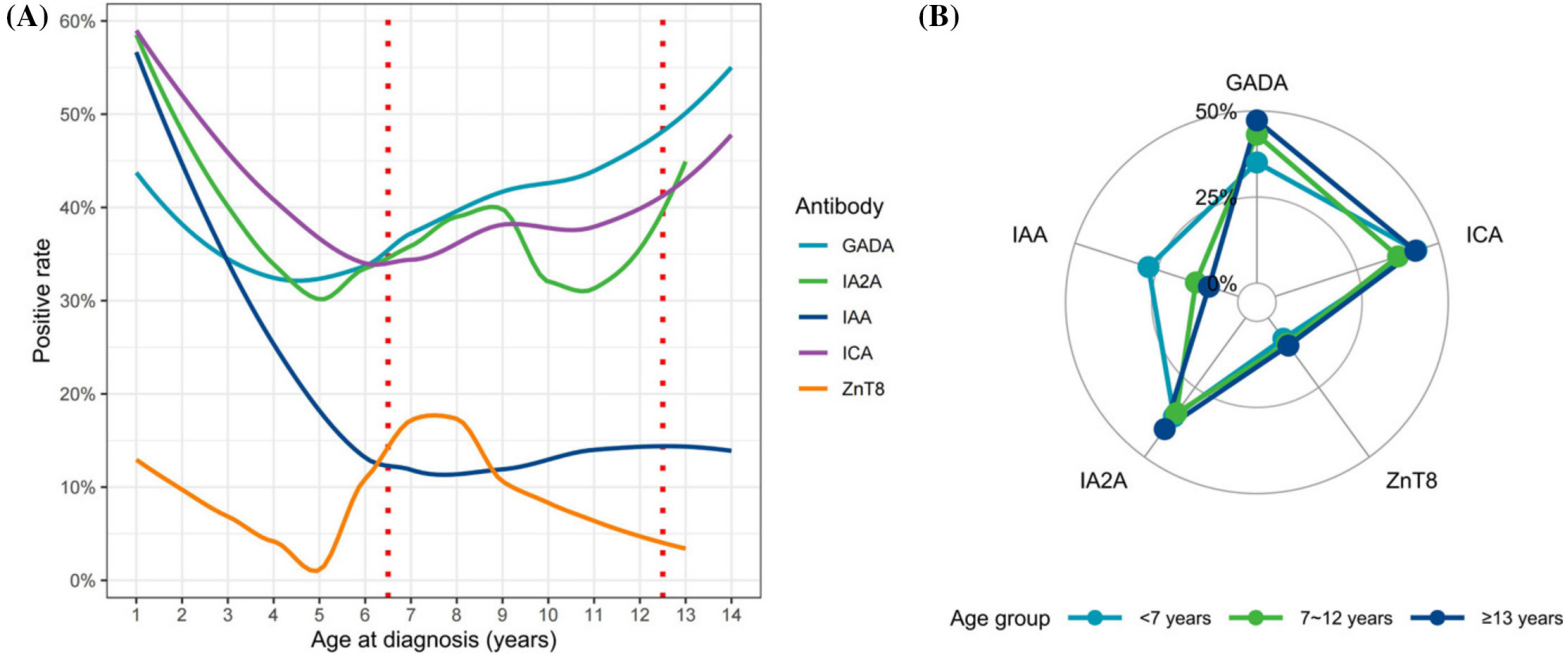
Positive rate of diabetes autoantibodies for different ages (A) and age groups (B). The smoothed lines were fitted by local polynomial regression. Red dotted lines denote the boundaries between age groups. GADA, glutamate decarboxylase antibody; IA2A, islet antigen 2 antibody; IAA, insulin autoantibody; ICA, islet cell antibody; ZnT8, zinc transporter 8 antibody.

In the cohort, the highest positivity rate was observed for ICA at 40%, followed by GADA at 39%, IA2A at 35%, IAA at 20%, and ZnT8 at 8%. We characterized the positive rate of the autoantibodies for different ages (Figure 3a). Both GADA and ICA showed U‐shaped curves, with the minimum positive rates at 4 and 6 years of age, respectively. The positive rate of IAA tended to decrease before 7 years of age and then stabilize. IA2A and ZnT8 showed similar but complex patterns. The positive rates of the autoantibodies in different age groups were visualized and compared in Figure 3b and Table 2. Positive rates for GADA and IAA exhibited heterogeneity among groups, with patients under 7 years of age demonstrating the lowest GADA positivity rate and the highest IAA positivity rate.

### 3.4 Other Clinical Characteristics

In our assessment of metabolic decompensation characteristics, we found no significant differences. However, significant distinctions emerged in thyroid function parameters, with the rates of TPOAb and TgAb positivity increasing with age. Furthermore, significant distinctions were noted in liver function parameters, as the prevalence of abnormal alanine aminotransferase (ALT) and alkaline phosphatase (ALP) levels rose with advancing age. Variations were also evident in creatine kinase‐MB (CK‐MB) levels.

## 4 Discussion

In this study, we comprehensively characterized the phenotypic spectrum of T1DM in terms of the age‐related endotypes in a Chinese population. The results showed age‐related heterogeneity in the characteristics of demographics, glucose metabolism, β‐cell autoimmunity, metabolic decompensation, thyroid function, and liver function. The T1DE1 endotype (patients < 7 years of age) was characterized by a shorter duration of symptoms before diagnosis. For glucose metabolism, it had the lowest fasting and postprandial C‐peptide and CGR and the highest postprandial glucose. It also had the highest IAA positive rate and CK‐MB level. The T1DE2 endotype (patients ≥ 13 years of age) was characterized by the highest level of HbA1c and the highest GADA‐positive rate. It has the highest proportion of patients with TPOAb and TgAb positivity, as well as the largest proportion of patients with abnormal liver function.

T1DM is caused by the destruction of pancreatic β‐cells, primarily mediated by T cells, which occurs when leukocytes (including CD8^+^, CD4^+^, and CD20^+^ cells) infiltrate and surround the pancreatic islets, a condition known as insulitis. Recent research has identified two distinct patterns of insulitis in individuals with newly diagnosed T1DM. These patterns, designated as CD20Hi and CD20Lo, differ primarily in the proportion of infiltrating CD20^+^ B cells [8]. This suggests that the aggressiveness of insulitis varies between these two forms, with patients exhibiting a CD20Hi profile experiencing a more rapid loss of β cells [18]. Furthermore, the histological subtypes of insulitis are associated with the age at diagnosis, namely, the T1DE1 and T1DE2 endotypes [9]. Our study found patients < 7 years of age had the poorest β‐cell function and the highest postprandial serum glucose levels, which is consistent with previous reports [19, 20]. This supports the previous notion that T1DE1 represents a more aggressive type of T1DM. Moreover, we compared the CGR levels and found that patients < 7 years of age had the lowest level of fasting and 2‐h CGR. It has been proposed that postprandial CGR is a reasonable estimate of pancreatic β‐cell area [21]. Our findings indicate that patients under the age of 7 have the smallest residual β‐cell area.

Despite having the worst β‐cell function at diagnosis, patients < 7 years of age had the lowest level of HbA1c compared to other age groups, which is consistent with the Finnish study [13]. This may be explained by the more aggressive nature of the disease. The acute onset and rapid progression from hyperglycemia to clinical T1DM is not fully captured by HbA1c measurements, which reflect average blood glucose levels over the preceding 2–3 months. Notably, in our cohort, 16 patients were diagnosed with fulminant T1DM according to the diagnostic criteria [22], with 15 of them in the T1DE1 group. Subgroup analysis was not conducted due to the limited number of patients. These patients exhibited acute symptom onset, rapid progression to DKA, and a significant decrease in C‐peptide levels within a short timeframe, suggesting the fulminant characteristic of the disease in this pediatric group.

The presence of autoantibodies to multiple islet autoantigens provides evidence for the autoimmune nature of T1DM. These autoantibodies do not directly cause the destruction of pancreatic β cells; rather, they arise as a result of β‐cell destruction mediated by T cells [23]. The positive rates of all autoantibodies were slightly higher than those reported in a Chinese study [19] but lower than those in a Finnish study [13]. Previous studies have shown that the prevalence of islet autoantibodies in the Asian population is significantly lower than that in the Caucasian population. This may be due to the heterogeneity of T1DM or the less sensitive assays [24]. A study of patients with T1DM found that the first antibodies to appear were IAA or GADA [23]. T1DE1 is characterized by the “IAA first” seroconversion, while T1DE2 is characterized by the “GADA first” seroconversion. Our findings also support this pattern of seroconversion. Figure 3a shows three age‐specific patterns of positivity: IA‐2A and ZnT8A, GADA, and IAA and ICA. IA‐2A and ZnT8 are considered surrogate markers for pancreatic β‐cell destruction, as they emerge directly before the onset of diabetes [23]. GADA, on the other hand, may not be a specific marker for pancreatic β‐cell destruction and may be associated with thyroid autoimmunity rather than insulitis in some cases [23]. As shown in Table 2, the rates of TPOAb and TgAb positivity were higher in the oldest age group, which is similar to GADA. The association between IAA and ICA has been studied less and requires further investigation.

Previous reports on DKA have shown contradictory results. While most studies have demonstrated that younger children are at a higher risk for DKA at diagnosis [25], the Finnish study showed that DKA was most common in the oldest age group [13]. In our study, the risk for DKA at diagnosis was similar for all three age groups. On the one hand, the extent of metabolic decompensation at diagnosis may reflect the underlying disease process, where younger patients may be more aggressive. On the other hand, it may be influenced by diagnostic delay, as evidenced by the higher HbA1c levels and longer duration of symptoms observed in older children. As a result of this combined effect, the risk of DKA may vary in different study settings.

Autoimmune thyroid disorders are the most prevalent immunological diseases in patients with T1DM [26], and T1DE2 is more frequently associated with thyroid autoimmunity [27]. Our results also support this association. However, it is still unclear whether this association is due to the confounding effect of age or if there is a pathological link. Age may function as a proxy for changes in immune and metabolic function, which impact both T1DM and thyroid autoimmunity [28]. While previous studies have primarily focused on association, future studies that explore causation may provide greater insight into the pathogenesis of the disease.

In addition to the two endotypes we evaluated, there are other T1DM, namely T1DE3–5, which display T2DM‐like phenotypes [29], and T1DE6, which represents T1DM induced through SARS‐CoV‐2 infection [30]. Further understanding of the endotypes may provide more evidence for the precise treatment of T1DM. The current classification system for T1DM is inadequate in reflecting the heterogeneity of the disease and, therefore, cannot inform precise management strategies. Recently, increased awareness of disease heterogeneity and the consequent necessity for personalized therapy has prompted growing interest in the endotype concept across various disease domains. The notion of T1DM endotypes was initially proposed based on pancreatic histopathology. However, the concept of T1DM endotypes remains a topic of debate, and further evidence is needed to support their existence. Advances have been made regarding precise interventions: T1DE1 may respond better to immunotherapies targeting specific immune cell subsets, such as rituximab or teplizumab, while T1DE2 may respond more favorably to GAD‐alum therapy [2]. Nevertheless, gaps remain in the evidence base concerning clinical manifestations. Prior research on T1DE1 and T1DE2 was primarily based on histopathological analyses of pancreatic samples. There is a dearth of phenotypic evidence, particularly regarding glucose metabolism, thyroid function, and other organ functions across age groups. The objective of this study was to comprehensively investigate the phenotypic heterogeneity of T1DM concerning age‐related endotypes. This study is particularly relevant for describing the phenotypic manifestations of T1DM endotypes and for validating the hypothesis that findings from histopathological analyses can be translated into phenotypic manifestations.

We acknowledge the limitations of our study. First, it is a single‐center study and lacks general representativeness. Second, due to the cross‐sectional nature of the study, we were unable to analyze treatment responses or long‐term prognosis. Our analysis was primarily focused on a comprehensive evaluation of the phenotypic spectrum at diagnosis. We recognize this limitation and plan to include follow‐up data in future work to enhance our understanding of the clinical outcomes associated with the identified endotypes. In addition, the data were collected from the pediatric department, which may not include all T1DM patients diagnosed after the age of 16, resulting in a smaller sample size for the ≥ 13 years age group.

## 5 Conclusion

Our study shows that there is phenotypic heterogeneity at T1DM diagnosis that is specific to age, including demographics, glucose metabolism, β‐cell autoimmunity, thyroid function, and liver function. These results suggest that there may be disease endotypes related to different age groups. Further study of these endotypes may provide more evidence for the precise treatment of T1DM.

## Author Contributions

Wei Gu conceived and designed the study. Qiaoli Zhou, Xueqin Zheng, and Chenguang Ma collected the data. Qiaoli Zhou conducted the data analysis. Qiaoli Zhou and Wei Gu contributed to the interpretation of the data. Qiaoli Zhou, Xueqin Zheng, and Chenguang Ma drafted the manuscript, while Wei Gu provided critical revisions. All authors gave final approval of the version to be published and agreed to be accountable for all aspects of the work.

## Ethics Statement

The study was reviewed and approved by the Children's Hospital of Nanjing Medical University (approval number: 202405001‐1) and conducted in accordance with the principles outlined in the Declaration of Helsinki.

## Conflicts of Interest

The authors declare no conflicts of interest.

## Supporting information


**Figure S1.** The overall workflow in the retrospective cohort construction. Data were extracted from the electronic medical records of all children who were reported to have new‐onset diabetes at the Children’s Hospital of Nanjing Medical University from 2010 to 2023. Abbreviations: DM, Diabetes mellitus; T1DM, Type 1 diabetes mellitus; T2DM, Type 2 diabetes mellitus.

## Data Availability

The data used in this study is confidential and subject to data protection regulations. Due to the sensitive nature of the data, it is not publicly available.
