# Supplementary material for: Phenotypic Spectrum at Diagnosis of Age‐Related Endotypes of Type 1 Diabetes Mellitus: A Cross‐Sectional Study in China
Source: J Diabetes. 2025 Jun 12;17(6):e70111. doi: 10.1111/1753-0407.70111 (PMC12159689; doi:10.1111/1753-0407.70111)
Supplement: Supplementary file 1 — Figure S1. The overall workflow in the retrospective cohort construction. Data were extracted from the electronic medical records of all children who were reported to have new‐onset diabetes at the Children’s Hospital of Nanjing Medical University from 2010 to 2023. Abbreviations: DM, Diabetes mellitus; T1DM, Type 1 diabetes mellitus; T2DM, Type 2 diabetes mellitus. [file JDB-17-e70111-s001.docx]

**Supplementary data**

**Supplementary Figure 1 legend**

**Supplementary Figure 1** The overall workflow in the retrospective cohort construction. Data were extracted from the electronic medical records of all children who were reported to have new-onset diabetes at the Children's Hospital of Nanjing Medical University from 2010 to 2023. Abbreviations: DM, Diabetes mellitus; T1DM, Type 1 diabtes mellitus; T2DM, Type 2 diabtes mellitus.

**
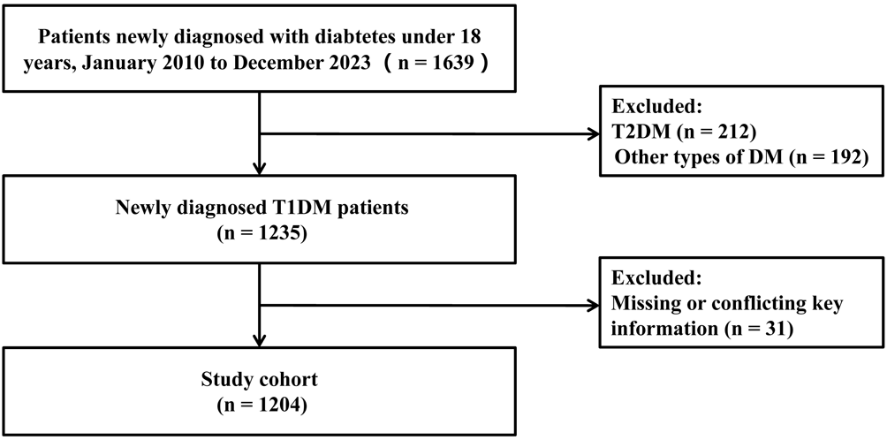
**
